# A Case of an Inflamed Pericardial Cyst As the Cause of Acute Onset Chest Pain in a Young Patient and Review of the Literature

**DOI:** 10.7759/cureus.23961

**Published:** 2022-04-08

**Authors:** Andreas Kyvetos, Stefani Panayiotou, Panagiota Voukelatou, Gerasimos Papavasileiou, Ioannis Vrettos

**Affiliations:** 1 2nd Department of Internal Medicine, General and Oncology Hospital of Kifissia “Agioi Anargyroi”, Athens, GRC; 2 Department of Thoracic Surgery, General Oncological Hospital of Kifissia “Agioi Anargyroi”, Athens, GRC

**Keywords:** pain, chest, acute, pericardial cyst, inflamed

## Abstract

Among the extensive variety of disorders that can cause chest pain are the complicated pericardial cysts, pathological entities that otherwise are asymptomatic. Here, we describe a 34-year-old male patient with a symptomatic pericardial cyst presenting at the emergency department with acute chest pain that woke him up about six hours prior to presentation. The work up for his acute chest pain revealed a well-defined, fluid-filled, rounded mass next to the pericardium on the right cardiophrenic angle and increased acute phase reactants. The cyst was surgically removed and the biopsy showed signs of intense inflammatory infiltration with negative culture of the fluid. The patient received intravenous antibiotics for two weeks with complete resolution of the symptoms and remained asymptomatic for about two months after surgical excision. Among other symptoms that may be induced from the presence of a pericardial cyst, the acute onset of chest pain, in this otherwise benign condition, probably indicates the existence of a complication such as rupture, inflammation, or hemorrhage. Careful exclusion of other etiologies of chest pain is mandatory as the surgical excision of a complicated pericardial cyst remains the only therapeutic option.

## Introduction

The evaluation of patients presenting with chest pain to the emergency department is challenging. As chest pain may be caused by numerous disorders, emergency physicians have to rule out life-threatening conditions through a systematic approach. Medical history, physical examination, electrocardiogram, laboratory examinations, imaging modalities, and decision algorithms are implemented to formulate an accurate diagnosis in a short time frame [1]. Among the extensive variety of disorders that can cause chest pain are the complicated pericardial cysts, pathological entities that otherwise are asymptomatic [2]. 

Here, we describe a young male patient with a symptomatic pericardial cyst presenting at the emergency department with acute chest pain.

## Case presentation

A 34-year-old Caucasian male presented at the emergency room because of acute chest pain that woke him up about six hours prior to presentation. The pain was worse when the patient was lying down and almost absent when he was standing up. He had a medical history of gastroesophageal reflux disease and he was a smoker (five packs per year) with no alcohol abuse. He was not taking any medication. The clinical examination of the patient was unremarkable with no pericardial rub or extra heart sounds.

**Figure 1:**
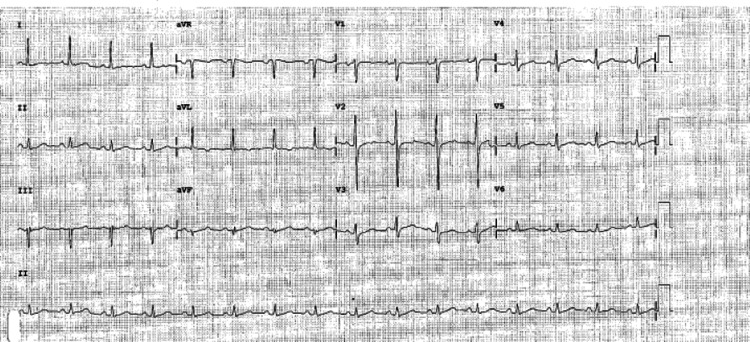
ECG on admission

The ECG on admission showed: sinus rhythm, 1:1 conduction of about 90 bpm, left axis deviation, and T wave inversion on augmented vector left (aVL) (Figure 1).

**Figure 2:**
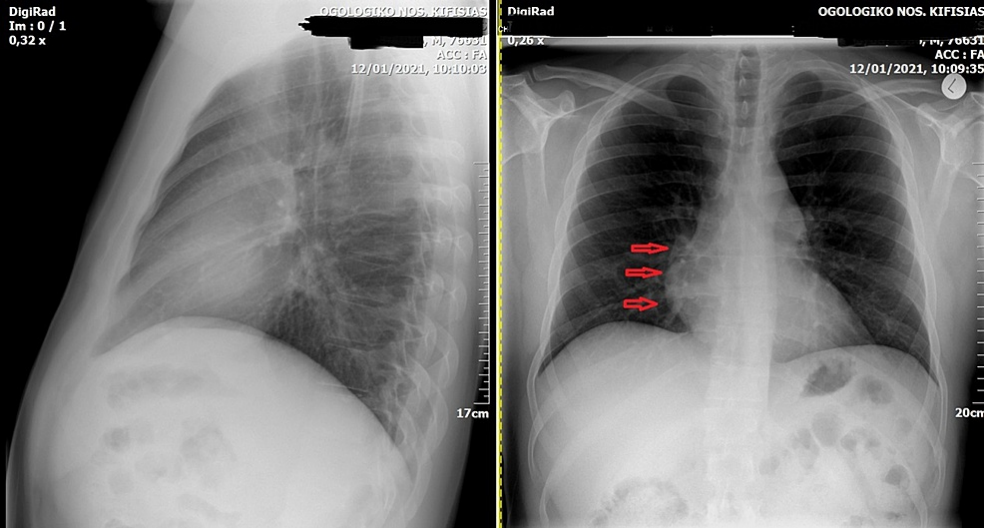
Lateral (left) and posteroanterior (right) chest radiograph showing the lesion (arrow) in the right cardio-phrenic angle.

A chest plain radiography showed a lesion silhouetting the cardiac border in the right cardio-phrenic angle (Figure 2).

**Figure 3:**
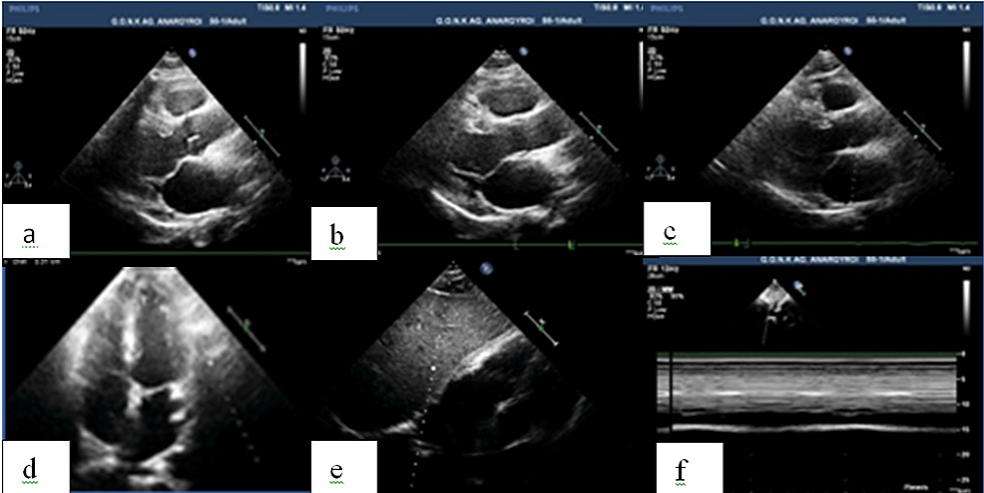
Cardiac ultrasound. Parasternal long axis view (a,b,c), apical 4 view (d), and subcostal view (e,f).

A cardiac ultrasound was performed that showed an ejection fraction of 60%, a mild mitral regurgitation, a mildly increased left atrial volume index of 35ml/m^2^ (normal range: < 34ml/m^2^ ), and no signs of pericardial effusion. Notably, the pericardial cyst was not visible by the transthoracic ultrasound (Figure 3.)

**Figure 4:**
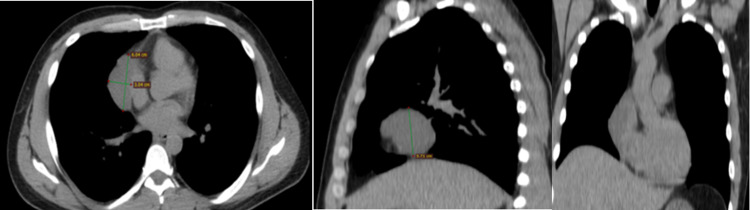
Chest CT demonstrating the pericardial cyst.

Initial laboratory analyses showed nothing pathological except of mild elevation in C-reactive protein (CRP) and erythrocyte sedimentation rate (ESR) levels: white blood cell count (WBC) = 10.55 x 10^3^ /μl (normal range: 4.0-11.0x10^3^ /μl), hematocrit (HCT) = 44.3% (normal range: 37-48%), hemoglobin (Hb) = 14.8 gr/dl (normal range: 12-16gr/dl ), ESR = 27mm/h (normal range: 2-20mm/h), creatinine (Cr) = 1.0 mg/dl (normal range: 0.6-1.4mgr/dl), CRP = 1.24 mg/dl (normal range: < 0.5mgr/dl), D-dimers < 168 μg/l (normal range: < 500μg/dl), first high-sensitivity troponin-Ι = 5.3 pg/ml (normal range: < 11.6 pg/ml), and second high-sensitivity troponin-Ι after 6 hours = 4 pg/ml. The subsequent chest CT scan showed a well-defined, fluid-filled, rounded mass next to the pericardium on the right cardiophrenic angle, measuring 6.0 cm × 3 cm × 4.3 cm (Figure 4).

**Figure 5:**
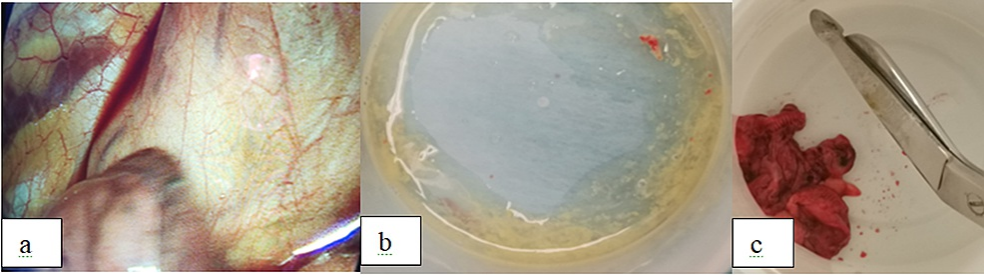
Perioperative view of the pericardial cyst (a). The fluid that was drained by the pericardial cyst (b). Small pieces of the pericardial cyst that were removed and sent for biopsy (c).

The patient was started on intravenous ceftriaxone 2 g once daily empirically and analgesics. In the first 12 hours, the pain increased and CRP was elevated at 9 mg/dl. A second cardiac ultrasound was performed with no change. At this time, the admission to the surgery room to underwent a video-assisted thoracic surgery (VATS) was decided. Perioperatively, the pericardial cyst was inflamed (Figure 5). The pericardial cyst was drained and the fluid was cultured. The cyst was removed and sent for biopsy. The cultures of the fluid were negative while the biopsy showed a pericardial cyst with signs of intense inflammatory infiltration. Moreover, blood and urine cultures taken before the administration of antibiotics and polymerase chain reaction (PCR) for coronavirus disease 2019 (COVID-19) were negative. Mantoux test, Quantiferon test, autoantibodies, and rheumatoid factor were also negative.

The patient became febrile soon after the surgery. Meropenem 2 g intravenous, three times daily and vancomycin 1 g intravenous, twice daily were administered. The fever lasted for around five days and the patient was discharged 14 days later, with no complication other than mild pleural effusion. Weekly follow up with ECG and ultrasound had shown nothing abnormal. About one month after the surgery, a chest CT was performed that revealed no signs of pericardial cyst or pleural effusion. A cardiac MRI was booked but because of COVID-19, it has been postponed. The patient remained asymptomatic for about two months after surgical excision, but after that, he could not be traced for follow up.

## Discussion

Pericardial cysts are rare entities with an incidence of 1 in 100,000 people. They are usually congenital in origin and they have to be differentiated from diverticula that may have similar radiological appearance. Usually, they are pictured as an isolated cystic shadow adjacent to the heart, most often in the right cardiophrenic angle. The majority of them are detected incidentally in a chest plain radiography made for other reasons [2]. For the management of asymptomatic pericardial cysts, non-specific treatment is recommended. Serial follow-up monitoring with CT has been proposed as an option for the evaluation of these cysts [3,4]. However, data regarding the safety of this practice are lacking [2]. On the other hand, percutaneous aspiration of the cyst as a first-line therapeutic approach and VATS or surgical resection as second-line choices were recommended for symptomatic pericardial cysts [5]. The presence of symptoms suggests compression of an adjacent structure or the development of a complication such as inflammation, hemorrhage, or rupture of the cyst [2]. 

Several symptoms and clinical pictures have been reported to bibliography associated with either the cyst itself or the presence of a complication including: chronic cough [6], recurrent syncope [7], pneumonia [8,9], shortness of breath [10,11] or dyspnea [3,11], palpitations [3,9,11], right-sided heart failure [12,13], atrial fibrillation [14], sudden death [15], chest discomfort or fullness [11], and chest pain [3,10,11,16-20].

In a series of 103 patients, chest pain has been reported as the most common symptom, followed by dyspnea. However, the etiology of chest pain, its characteristics or whether it is acute or chronic are not mentioned [3]. In some previous published cases, pain was attributed to the size of the cyst itself and it had chronic character [11,16]. In other cases, with acute onset of chest pain or exacerbation of chronic chest pain, the cause was hemorrhage into the cyst [19], inflammation of a cyst [17], or the rupture of an inflamed cyst [18,20]. In all the cases of inflamed cysts, no specific cause of this inflammation process has been ascertained. In one more case where the pain was acute and was attributed to the size of the cyst, no cytology result of the resected cyst was referred [10].

## Conclusions

Pericardial cyst is a rare entity, chest pain is the most common clinical manifestation. Acute onset of chest pain, in this otherwise benign condition, indicates probably the existence of a complication such as rupture, inflammation, or hemorrhage. Careful exclusion of other etiologies of chest pain is mandatory as the surgical excision of a complicated pericardial cyst remains the only therapeutic option. So, the benefits of this invasive approach should be weighed against the procedural risks. 
